# 5-Bromo-2-meth­oxy-4-{[(4-meth­oxy­phen­yl)imino]­meth­yl}phenol monohydrate

**DOI:** 10.1107/S1600536811054742

**Published:** 2012-01-07

**Authors:** Cheng-Gong Mao, Shuang-Shuang Wang, Deng-Cheng Su, Shao-Song Qian

**Affiliations:** a School of Life Sciences, ShanDong University of Technology, ZiBo 255049, People’s Republic of China

## Abstract

The crystal structure of the title compound, C_15_H_14_BrNO_3_·H_2_O, has a *trans* configuration about the central C=N double bond. An intra­molecular O—H⋯O hydrogen bond occurs in the main mol­ecule. The crystal packing is stabilized by strong O—H⋯O and O—H⋯N hydrogen bonds.

## Related literature

For related structures, see: Shao *et al.* (2004 ▶); Cheng *et al.* (2005) ▶.
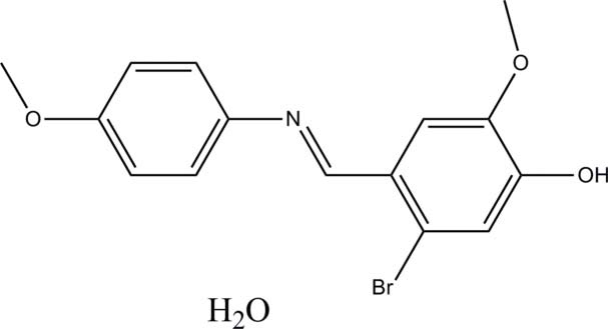


## Experimental

### 

#### Crystal data

C_15_H_14_BrNO_3_·H_2_O
*M* *_r_* = 354.20
Orthorhombic, 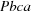

*a* = 13.992 (4) Å
*b* = 7.219 (2) Å
*c* = 30.232 (9) Å
*V* = 3053.5 (15) Å^3^
*Z* = 8
Mo *K*α radiation
μ = 2.71 mm^−1^
*T* = 296 K
0.23 × 0.12 × 0.08 mm

#### Data collection

Bruker APEXII CCD diffractometer
Absorption correction: multi-scan (*SADABS*; Bruker, 2004 ▶) *T*_min_ = 0.575, *T*_max_ = 0.813
20409 measured reflections
2836 independent reflections
1831 reflections with *I* > 2σ(*I*)
*R*_int_ = 0.082

#### Refinement

*R*[*F* ^2^ > 2σ(*F* ^2^)] = 0.045
*wR*(*F* ^2^) = 0.113
*S* = 1.02
2836 reflections
201 parameters
3 restraints
H atoms treated by a mixture of independent and constrained refinement
Δρ_max_ = 0.39 e Å^−3^
Δρ_min_ = −0.46 e Å^−3^

### 

Data collection: *APEX2* (Bruker, 2004 ▶); cell refinement: *SAINT* (Bruker, 2004 ▶); data reduction: *SAINT*; program(s) used to solve structure: *SHELXS97* (Sheldrick, 2008 ▶); program(s) used to refine structure: *SHELXL97* (Sheldrick, 2008 ▶); molecular graphics: *SHELXTL* (Sheldrick, 2008 ▶); software used to prepare material for publication: *SHELXTL*.

## Supplementary Material

Crystal structure: contains datablock(s) global, I. DOI: 10.1107/S1600536811054742/qm2045sup1.cif

Structure factors: contains datablock(s) I. DOI: 10.1107/S1600536811054742/qm2045Isup2.hkl

Supplementary material file. DOI: 10.1107/S1600536811054742/qm2045Isup3.cml

Additional supplementary materials:  crystallographic information; 3D view; checkCIF report

## Figures and Tables

**Table 1:** Hydrogen-bond geometry (Å, °)

*D*—H⋯*A*	*D*—H	H⋯*A*	*D*⋯*A*	*D*—H⋯*A*
O1—H1⋯O2	0.82	2.24	2.683 (4)	114
O1—H1⋯O4	0.82	1.92	2.668 (5)	152
O4—H1*W*⋯O1^i^	0.85 (3)	2.03 (4)	2.880 (5)	176 (6)
O4—H2*W*⋯N1^ii^	0.85 (4)	2.05 (4)	2.903 (5)	176 (3)

Symmetry codes: (i) 
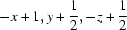
; (ii) 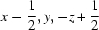.

**Fig. 1.**
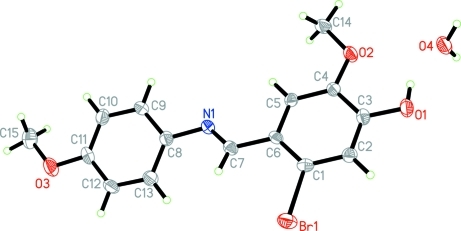
The structure of (I), showing the atom-labelling scheme. Displacement ellipsoids are drawn at the 30% probability level.

## supplementary crystallographic information

### Comment

The 2-bromo-4-hydroxy-5-methoxybenzaldehyde can react with organic amines to form a range of Schiff bases. Schiff base compounds are well known for their wide range of biological activities and have contributed to the development of coordination chemistry related to catalysis, enzymatic reactions, magnetism and molecular architecture (Zhu *et al.*, 2005). Here, We report one of the schiff bases which is structrually characterized to promote the development of coordination chemistry. The title compound displays a trans-configuration with respect to the C(7)=N(1) double bond. The compound crystallized in the orthorhombic system with one title compound molecule and a water molecule in the asymmetric unit. There is a π-π interaction (symmetry code: 3/2-X,1/2+Y,Z) [centroid-centroid distance =3.758 (3) Å ] There are also O(4)—H(1W)···O(1) and O(4)—H(2W)···N(1) hydrogen bonds with symmetry codes (1-x,1/2+y,1/2-z)and (-1/2+x,y,1/2-z) respectively (Figure 2 and table 1).

### Experimental

The 2-bromo-4-hydroxy-5-methoxybenzaldehyde (0.1155 g) and 4-methoxyaniline (0.0616 g) were dissolved in methanol (20 mL) and reacted at room temperature for 30 mins to give a clear solution. The solution after standingin in air for 5 days gave yellow block-shaped single crystals at the bottom of the reaction vessel which were suitable for X-ray diffraction analysis.

### Refinement

All H atoms were placed in geometrical positions and constrained to ride on their parent atoms with C—H distances in the range 0.93–0.96 Å, They were treated as riding atoms, with *U*_iso_(H) = k*U*_eq_(C), where k = 1.5 for methyl and 1.2 for all other H atoms.

### Crystal data

**Table:**

C_15_H_14_BrNO_3_·H_2_O	*D*_x_ = 1.541 Mg m^−^^3^
*M**_r_* = 354.20	Mo *K*α radiation, λ = 0.71073 Å
Orthorhombic, *P**b**c**a*	Cell parameters from 2836 reflections
*a* = 13.992 (4) Å	θ = 2.7–25.5°
*b* = 7.219 (2) Å	µ = 2.71 mm^−^^1^
*c* = 30.232 (9) Å	*T* = 296 K
*V* = 3053.5 (15) Å^3^	Block, yellow
*Z* = 8	0.23 × 0.12 × 0.08 mm
*F*(000) = 1440	

### Data collection

**Table:**

Bruker APEXII CCD diffractometer	2836 independent reflections
Radiation source: fine-focus sealed tube	1831 reflections with *I* > 2σ(*I*)
graphite	*R*_int_ = 0.082
φ and ω scans	θ_max_ = 25.5°, θ_min_ = 2.7°
Absorption correction: multi-scan (*SADABS*; Bruker, 2004)	*h* = −16→16
*T*_min_ = 0.575, *T*_max_ = 0.813	*k* = −8→8
20409 measured reflections	*l* = −36→33

### Refinement

**Table:**

Refinement on *F*^2^	Primary atom site location: structure-invariant direct methods
Least-squares matrix: full	Secondary atom site location: difference Fourier map
*R*[*F*^2^ > 2σ(*F*^2^)] = 0.045	Hydrogen site location: inferred from neighbouring sites
*wR*(*F*^2^) = 0.113	H atoms treated by a mixture of independent and constrained refinement
*S* = 1.01	*w* = 1/[σ^2^(*F*_o_^2^) + (0.0442*P*)^2^ + 3.2392*P*] where *P* = (*F*_o_^2^ + 2*F*_c_^2^)/3
2836 reflections	(Δ/σ)_max_ = 0.001
201 parameters	Δρ_max_ = 0.39 e Å^−^^3^
3 restraints	Δρ_min_ = −0.46 e Å^−^^3^

### Special details

**Table:**

Geometry. All esds (except the esd in the dihedral angle between two l.s. planes) are estimated using the full covariance matrix. The cell esds are taken into account individually in the estimation of esds in distances, angles and torsion angles; correlations between esds in cell parameters are only used when they are defined by crystal symmetry. An approximate (isotropic) treatment of cell esds is used for estimating esds involving l.s. planes.
Refinement. Refinement of F^2^ against ALL reflections. The weighted R-factor wR and goodness of fit S are based on F^2^, conventional R-factors R are based on F, with F set to zero for negative F^2^. The threshold expression of F^2^ > 2sigma(F^2^) is used only for calculating R-factors(gt) etc. and is not relevant to the choice of reflections for refinement. R-factors based on F^2^ are statistically about twice as large as those based on F, and R- factors based on ALL data will be even larger.

### Fractional atomic coordinates and isotropic or equivalent isotropic displacement parameters (Å^2^)

**Table:**

	*x*	*y*	*z*	*U*_iso_*/*U*_eq_	
Br1	0.68925 (3)	0.04514 (7)	0.438691 (15)	0.05101 (19)	
C1	0.7051 (3)	0.1140 (6)	0.37837 (13)	0.0341 (10)	
C2	0.6237 (3)	0.1493 (6)	0.35403 (14)	0.0377 (10)	
H2	0.5640	0.1407	0.3674	0.045*	
C3	0.6305 (3)	0.1969 (6)	0.31041 (14)	0.0367 (10)	
C4	0.7199 (3)	0.2112 (6)	0.29032 (14)	0.0332 (10)	
C5	0.8008 (3)	0.1774 (6)	0.31504 (13)	0.0324 (9)	
H5	0.8604	0.1865	0.3016	0.039*	
C6	0.7955 (3)	0.1300 (6)	0.35947 (13)	0.0324 (9)	
C7	0.8840 (3)	0.1008 (6)	0.38482 (14)	0.0365 (10)	
H7	0.8803	0.0479	0.4128	0.044*	
C8	1.0489 (3)	0.1209 (6)	0.39542 (14)	0.0372 (10)	
C9	1.1347 (3)	0.1063 (6)	0.37270 (14)	0.0450 (12)	
H9	1.1344	0.1048	0.3419	0.054*	
C10	1.2206 (3)	0.0938 (6)	0.39508 (16)	0.0469 (12)	
H10	1.2773	0.0814	0.3793	0.056*	
C11	1.2226 (3)	0.0996 (6)	0.44019 (15)	0.0448 (11)	
C12	1.1378 (3)	0.1129 (7)	0.46330 (15)	0.0471 (12)	
H12	1.1387	0.1152	0.4941	0.057*	
C13	1.0520 (3)	0.1227 (6)	0.44131 (14)	0.0432 (11)	
H13	0.9954	0.1307	0.4573	0.052*	
C14	0.8055 (3)	0.2671 (7)	0.22380 (14)	0.0503 (12)	
H14A	0.8448	0.3609	0.2371	0.075*	
H14B	0.7940	0.2979	0.1934	0.075*	
H14C	0.8377	0.1498	0.2255	0.075*	
C15	1.3933 (3)	0.1070 (7)	0.44297 (17)	0.0636 (15)	
H15A	1.4020	−0.0007	0.4248	0.095*	
H15B	1.4440	0.1140	0.4643	0.095*	
H15C	1.3941	0.2159	0.4247	0.095*	
H1W	0.510 (4)	0.471 (4)	0.2082 (17)	0.08 (2)*	
H2W	0.511 (3)	0.293 (5)	0.1846 (12)	0.068 (18)*	
N1	0.9655 (2)	0.1462 (5)	0.36950 (11)	0.0368 (8)	
O1	0.54833 (18)	0.2278 (5)	0.28775 (10)	0.0489 (8)	
H1	0.5610	0.2520	0.2619	0.073*	
O2	0.71757 (19)	0.2562 (4)	0.24658 (9)	0.0446 (8)	
O3	1.3042 (2)	0.0951 (5)	0.46530 (11)	0.0578 (9)	
O4	0.5312 (2)	0.3608 (6)	0.20578 (12)	0.0569 (9)	

### Atomic displacement parameters (Å^2^)

**Table:**

	*U*^11^	*U*^22^	*U*^33^	*U*^12^	*U*^13^	*U*^23^
Br1	0.0582 (3)	0.0610 (3)	0.0338 (3)	−0.0061 (2)	0.0028 (2)	0.0022 (2)
C1	0.045 (2)	0.027 (2)	0.031 (2)	−0.0039 (18)	0.0047 (18)	−0.0021 (18)
C2	0.034 (2)	0.042 (3)	0.038 (3)	−0.0044 (19)	0.0021 (19)	−0.004 (2)
C3	0.034 (2)	0.033 (3)	0.043 (3)	0.0006 (18)	−0.0071 (19)	−0.002 (2)
C4	0.036 (2)	0.031 (2)	0.033 (2)	0.0013 (18)	−0.0013 (18)	−0.0012 (19)
C5	0.029 (2)	0.035 (2)	0.034 (2)	−0.0016 (17)	0.0002 (17)	−0.0022 (19)
C6	0.039 (2)	0.031 (2)	0.027 (2)	0.0005 (18)	−0.0050 (18)	−0.0033 (19)
C7	0.045 (2)	0.032 (3)	0.032 (2)	0.0017 (19)	−0.007 (2)	−0.0027 (19)
C8	0.036 (2)	0.037 (3)	0.038 (3)	0.0013 (18)	−0.0040 (19)	−0.001 (2)
C9	0.049 (3)	0.056 (3)	0.030 (2)	0.009 (2)	−0.002 (2)	0.003 (2)
C10	0.036 (2)	0.053 (3)	0.051 (3)	0.008 (2)	0.001 (2)	0.005 (2)
C11	0.047 (3)	0.043 (3)	0.045 (3)	0.001 (2)	−0.015 (2)	0.007 (2)
C12	0.050 (3)	0.063 (3)	0.028 (2)	−0.002 (2)	−0.005 (2)	0.001 (2)
C13	0.042 (2)	0.048 (3)	0.040 (3)	−0.003 (2)	−0.001 (2)	0.002 (2)
C14	0.049 (3)	0.070 (4)	0.032 (3)	−0.005 (3)	0.004 (2)	0.005 (2)
C15	0.046 (3)	0.064 (4)	0.081 (4)	−0.002 (2)	−0.009 (3)	0.009 (3)
N1	0.0359 (19)	0.042 (2)	0.032 (2)	0.0020 (16)	−0.0056 (15)	−0.0016 (17)
O1	0.0317 (16)	0.068 (2)	0.0469 (19)	0.0015 (14)	−0.0065 (14)	0.0111 (18)
O2	0.0406 (16)	0.060 (2)	0.0329 (17)	0.0006 (14)	−0.0063 (13)	0.0115 (15)
O3	0.0430 (18)	0.078 (3)	0.052 (2)	−0.0042 (16)	−0.0152 (16)	0.0115 (18)
O4	0.057 (2)	0.061 (3)	0.052 (2)	0.0146 (19)	−0.0209 (17)	−0.010 (2)

### Geometric parameters (Å, °)

**Table:**

Br1—C1	1.903 (4)	C10—C11	1.365 (6)
C1—C2	1.380 (5)	C10—H10	0.9300
C1—C6	1.393 (5)	C11—O3	1.371 (5)
C2—C3	1.366 (5)	C11—C12	1.381 (6)
C2—H2	0.9300	C12—C13	1.374 (6)
C3—O1	1.356 (4)	C12—H12	0.9300
C3—C4	1.395 (5)	C13—H13	0.9300
C4—O2	1.362 (5)	C14—O2	1.413 (5)
C4—C5	1.378 (5)	C14—H14A	0.9600
C5—C6	1.388 (5)	C14—H14B	0.9600
C5—H5	0.9300	C14—H14C	0.9600
C6—C7	1.471 (5)	C15—O3	1.421 (5)
C7—N1	1.275 (5)	C15—H15A	0.9600
C7—H7	0.9300	C15—H15B	0.9600
C8—C9	1.386 (5)	C15—H15C	0.9600
C8—C13	1.388 (6)	O1—H1	0.8200
C8—N1	1.418 (5)	O4—H1W	0.855 (18)
C9—C10	1.383 (6)	O4—H2W	0.853 (18)
C9—H9	0.9300		
			
C2—C1—C6	121.0 (4)	C11—C10—H10	119.8
C2—C1—Br1	117.6 (3)	C9—C10—H10	119.8
C6—C1—Br1	121.4 (3)	C10—C11—O3	124.7 (4)
C3—C2—C1	120.3 (4)	C10—C11—C12	119.3 (4)
C3—C2—H2	119.9	O3—C11—C12	115.9 (4)
C1—C2—H2	119.9	C13—C12—C11	120.7 (4)
O1—C3—C2	118.0 (4)	C13—C12—H12	119.7
O1—C3—C4	121.9 (4)	C11—C12—H12	119.7
C2—C3—C4	120.1 (4)	C12—C13—C8	120.6 (4)
O2—C4—C5	126.1 (4)	C12—C13—H13	119.7
O2—C4—C3	114.8 (3)	C8—C13—H13	119.7
C5—C4—C3	119.2 (4)	O2—C14—H14A	109.5
C4—C5—C6	121.7 (4)	O2—C14—H14B	109.5
C4—C5—H5	119.2	H14A—C14—H14B	109.5
C6—C5—H5	119.2	O2—C14—H14C	109.5
C5—C6—C1	117.8 (3)	H14A—C14—H14C	109.5
C5—C6—C7	119.6 (4)	H14B—C14—H14C	109.5
C1—C6—C7	122.6 (4)	O3—C15—H15A	109.5
N1—C7—C6	121.8 (4)	O3—C15—H15B	109.5
N1—C7—H7	119.1	H15A—C15—H15B	109.5
C6—C7—H7	119.1	O3—C15—H15C	109.5
C9—C8—C13	118.0 (4)	H15A—C15—H15C	109.5
C9—C8—N1	116.6 (4)	H15B—C15—H15C	109.5
C13—C8—N1	125.2 (4)	C7—N1—C8	120.2 (4)
C10—C9—C8	121.0 (4)	C3—O1—H1	109.5
C10—C9—H9	119.5	C4—O2—C14	117.7 (3)
C8—C9—H9	119.5	C11—O3—C15	117.8 (4)
C11—C10—C9	120.3 (4)	H1W—O4—H2W	119 (3)
			
C6—C1—C2—C3	1.4 (6)	C13—C8—C9—C10	0.0 (7)
Br1—C1—C2—C3	−179.1 (3)	N1—C8—C9—C10	175.6 (4)
C1—C2—C3—O1	179.1 (4)	C8—C9—C10—C11	−1.4 (7)
C1—C2—C3—C4	−0.4 (6)	C9—C10—C11—O3	−177.5 (4)
O1—C3—C4—O2	−0.7 (6)	C9—C10—C11—C12	1.8 (7)
C2—C3—C4—O2	178.8 (4)	C10—C11—C12—C13	−0.9 (7)
O1—C3—C4—C5	−179.7 (4)	O3—C11—C12—C13	178.5 (4)
C2—C3—C4—C5	−0.2 (6)	C11—C12—C13—C8	−0.5 (7)
O2—C4—C5—C6	−179.1 (4)	C9—C8—C13—C12	0.9 (7)
C3—C4—C5—C6	−0.2 (6)	N1—C8—C13—C12	−174.3 (4)
C4—C5—C6—C1	1.2 (6)	C6—C7—N1—C8	178.2 (4)
C4—C5—C6—C7	−178.1 (4)	C9—C8—N1—C7	156.8 (4)
C2—C1—C6—C5	−1.8 (6)	C13—C8—N1—C7	−28.0 (7)
Br1—C1—C6—C5	178.8 (3)	C5—C4—O2—C14	1.0 (6)
C2—C1—C6—C7	177.5 (4)	C3—C4—O2—C14	−178.0 (4)
Br1—C1—C6—C7	−2.0 (6)	C10—C11—O3—C15	7.8 (7)
C5—C6—C7—N1	11.5 (6)	C12—C11—O3—C15	−171.6 (4)
C1—C6—C7—N1	−167.7 (4)		

### Hydrogen-bond geometry (Å, °)

**Table:**

*D*—H···*A*	*D*—H	H···*A*	*D*···*A*	*D*—H···*A*
O1—H1···O2	0.82	2.24	2.683 (4)	114
O1—H1···O4	0.82	1.92	2.668 (5)	152
O4—H1W···O1^i^	0.85 (3)	2.03 (4)	2.880 (5)	176 (6)
O4—H2W···N1^ii^	0.85 (4)	2.05 (4)	2.903 (5)	176 (3)

Symmetry codes: (i) −*x*+1, *y*+1/2, −*z*+1/2; (ii) *x*−1/2, *y*, −*z*+1/2.
